# New York Odontological Society

**Published:** 1895-12

**Authors:** 

**Affiliations:** New York Odontological Society


### Reports of Society Meetings.

### NEW YORK ODONTOLOGICAL SOCIETY.

A REGULAR meeting of the New York Odontological Society was held on Tuesday evening, October 15, 1895, at the New York Academy of Medicine, with the President, Dr. Northrop, in the chair.

The minutes of the last meeting were read and approved.

Dr. Frederick Peterson then presented a paper entitled "Deformities of the Hard Palate in Degenerates," which elicited the following discussion.

(For Dr. Peterson's paper, see page 719.)

### DISCUSSION.

*Professor Peirce*, of Philadelphia.—I desire to acknowledge that it has been a great pleasure to listen to the essayist's paper, and especially so because it is a very good omen in our profession to have a paper of this kind read before any dental society. It is evidence that we are broadening our opportunities, and are looking beyond the mechanical and financial part of our profession. The paper presents many sides, and it is difficult to discuss it, because the doctor has gone over the subject thoroughly, and leaves but little to criticise or to add. The first inquiry is, What is degeneration? The essayist answered that in his first paragraph. He said it was a morbid deviation from a typical standard. That is about the idea. We all know that Nature seldom makes two things alike,—indeed, never does. It is, therefore, very difficult for us to say to what extent this variation may take place in the development of any organ of the human family, and yet not be beyond the limits of normal or not be morbid. We are all familiar with a variety of palates. When they go to a certain extent we call them monstrosities, and when they become such we try to correct them and bring them as near as possible to the normal type for the advantage of the patient. In speaking of degenerates, the most unfortunate part of it is that all degenerates are congenital. They are victims, because the unconscious biology does not differ from conscious biology, and therefore a development that is influenced in embryo is a development, of course, far beyond the influence of the victim. They are therefore helpless and innocent victims, for the biological law of development is unyielding. The victim is here because he could not help himself, and that is the unfortunate part of degeneracy. The question that interests me more than anything else is to see if we can trace any cause for this deviation from the normal,—whether we can go back and get any indication whatever as to why this development has been abnormal and resulted in a victim who is an unfortunate member of society? I wish the doctor had gone a little further into histology, because he is evidently very competent, and given us some idea of just how changes take place, and what would be the influence that would be exerted on the developing tissue to bring the result. Let me illustrate: an illustration that I believe I got one day from Max Nordau's book. You know he wrote a remarkable work on degeneration, which has been criticised the world over. He throws a stone into the water: the impulse of that upon the fluid is in proportion to the condition of the fluid as well as the force of the stone. Then he states that, taking the cortical brain-cells, the impulse of nutrition is responded to in those cells in proportion as they are in a condition to receive. The inference drawn was, that if there is some habit of either parent that has placed those cells in an abnormally responsive condition, then we have either an arrest or a modification of healthy development, and so we may have what is called physical degeneration. The idea was carried out, giving some idea of how the physical degeneration took place; and, having the physical, we must have psychical degeneration as a result. One is dependent upon the other, and we cannot have one without the other.

Degeneration, as Dr. Peterson has said, may manifest itself in the shape of the head, in the size of the ear, in peculiarities of the eyes, nose, teeth, and palate. They are all simple expressions of abnormal nutrition, and abnormal nutrition because there has been some abnormal germ in the embryo. Another question that presents itself is, If these degenerates can reproduce, we are going to have a large class of them in society, very soon a species; but, fortunately, there is another biological law that comes to the rescue, and that is, that where we have a true degenerate, any germ of degeneracy in the individual will be increased probably in the first and second generation, but in the third generation, it is hoped, becomes sterile. It is stated that degenerates cannot reproduce beyond the third generation. This being so, there cannot be a race or species of degenerates. A true species would be normal in that it would reproduce like the parent. Degenerates do not produce like themselves. There is always an increased degradation in the reproduction until the third generation, when sterility shuts it off; so society is protected.

When the doctor closes the discussion, I hope he will give us his idea as to whether he thinks degenerates are a return to an ancestral condition, and whether in any one of these conditions which he has exhibited he thinks it is a retrogression to an ancestral type. It is an interesting question, whether there has been some form in lower normal life to which they are simply a return or reversion, or whether the abnormality is owing to some unresponsive condition of the cell due to the degenerate germ having been there implanted in embryo.

*Professor Abbott.*—I have to confess an inability to properly discuss a question of this importance. It is a question that I have never studied specially, although I have seen many cases of the kind which Dr. Peterson has spoken of in his paper. I have never studied it for its sake and for the sake of the human race. There is one point in connection with the subject, however, which I think the doctor has overlooked, which we, as dental surgeons, recognize. That is, that in almost every instance of abnormal development of the bones of the face abnormal development of the teeth is present. This condition of the dental organs probably dates, for its origin, as far back as the beginning of the formation of the palate. These anomalies indicate something wrong in the first beginning of the development of the child. The teeth have been overlooked by men in the study of this question. Not one of the models shown here this evening has a normal type of teeth.

It is a fact that all degenerates, if they attempt to propagate, as Dr. Peirce has said, become sterile and soon die out, provided they intermarry. Such families disappear entirely within a few generations. The question often arises, Are we really degenerating as a race in the world to-day? This question was discussed at some length in a criticism of Nordau's work by Charles A. Dana, of this city, in the June, 1895, *Forum.* He says that in 1880, from the statistics in this country, there were eighteen hundred and sixty-three lunatics to the million people, and eighteen hundred and fifty-three idiotic, and in 1890 sixteen hundred and ninety-seven lunatics and fifteen hundred and twenty-six idiots to the million. Making some allowance for possible mistakes in both of these cases, it would still indicate that there is not a general tendency to degeneration, mentally or physically. The condition of the palate which the doctor laid considerable stress upon, this enlargement or hyperostasis in the roof of the mouth, is a condition that I have seen more often than any other deformity, probably because the patients that we see are not the kind that he has been examining. The people he refers to, and the casts of whose mouths we have been invited to examine, are not the kind that go to a dentist to have their teeth attended to, so we cannot compare them. We have very little opportunity really for studying these abnormalities, unless we go with him into the lunatic asylums, or among idiots, and study the subject from the stand-point that he has done. It is with so much difficulty that we can study the question to any extent that we depend almost entirely for our knowledge of the subject upon gentlemen like the author of the paper. The better class of people are not, in my judgment, degenerating physically or mentally to any great or alarming extent, if at all.

*Dr. Ottolengui.*—I feel just a little foolish to-night, because I have brought with me a case full of models that seem to me to be rather out of place. I labored under a misconception of the subject. I understood that it was to be a discussion on cleft palate in its relation to degeneracy, and when I was asked by the chairman of the Executive Committee to bring some models with me, I agreed to do so. As long as I have them here, I will show them to you. They are from the collection of Dr. Kingsley.

There is one point that I would make now in connection with the models, and that is, whilst they are exceedingly abnormal mouths, they are casts from normal people. The patients are not degenerates in any sense of the word, so far as I know. They have not been convicted of crime, nor considered degenerate mentally. I would ask you to notice the marked similarity between many of the models, showing that even in abnormality there is a certain typical condition. Some of the models have instruments in them, which indicate the treatment which they have received, and one lot of casts represents children's mouths.

I would like to start by saying that I have recently been paying some little attention to criminal anthropology, and in that realm the most noted writer I think is Lombroso. I have made note of a few statistical figures which have a bearing upon our dental practice in connection with degenerates. Dr. Peirce has touched upon the subject of a return to the type of a degenerate ancestor. He asked whether that is the explanation of the abnormalities in connection with degenerates. Lombroso holds that it is, and that atavism explains everything. He shows that criminals who have a certain return to the physical ancestral type also have a definite moral turpitude. For example, a certain form of jaw is peculiar to murderers, and the murderer who is a born criminal, who murders for gain and not in the heat of passion, must be more or less typical of a brute. The distinction between killing for personal advantage, for food, and for gain is a demarcation which has been made by civilization. If you find that in the large proportion of these murderers there is a given shape of the jaw or head, or various portions of the body, all of which are more or less a return to the ancestral form, that is a very good point in favor of the atavistic theory. He says, in speaking of these cases, that by an enormous lower jaw is meant a jaw which, when considered in proportion to the skull, is much larger than in the normal man. The prognathus jaw is found in seven per cent. of criminals, twelve per cent. of assassins, and thirteen per cent. of profligates. As to anomalous teeth: sixteen per cent. of criminals, twenty-eight per cent. of profligates, and eight per cent. of normals; so that those of us who make our living out of the normal have only eight per cent. to draw from. Madame Tarnousky, who has been a student of the subject, finds that in Russia there are anomalous teeth in forty per cent. of homicides, fifty-one per cent. of thieves, and seventy-eight per cent. of profligates. At first you might imagine that anomalous teeth are more common in Russia than elsewhere, but she finds anomalous teeth in only two per cent. of normals. The peculiar political condition of Russia is such that there is a very small intergrade between the law-abiding and the law-breaking. It is either for the Czar or against the Czar. They incarcerate people for very much smaller crimes there than anywhere else.

A word about what these anomalies of teeth are. One anomaly of the teeth (and this is peculiar among murderers) is the very large and prominent canine, with the other teeth dwarfed. If you look at some of the skulls in the Museum here, you will see at once that with prominent canines and prominent jaws you have a return to the ancestral condition very well defined. You have a retrogression to the anthropoid ape, and it is natural that we should find it in the class of people who have least regard for the laws of humanity. One of this type is a woman who had killed seventeen infants, and murdered over forty people altogether. She had nearly all these stigmata. While it was perhaps well to hang her, there was not the slightest excuse for killing her, from the standpoint of justice. It was purely selfish to put her out of the way, because she was a born criminal, and simply followed her natural bent.

Thirty per cent. of profligates in Italy have dwarfed lateral incisors,—atrophy of the lateral incisors. That seems very peculiar. In connection with cleft palate, the essayist said he had found more cleft palates among normal people than among idiots; but Lombroso says a cleft palate is most common among profligates. He placed the figures at one and one half in a thousand among normal individuals; that would make three persons in two thousand. He finds as many as nine in one thousand among criminals, and fifteen in one thousand among profligates. We should be very careful in accepting statistics. The addition of one or two more individuals alters the percentage. While Lombroso always speaks of *congenital* cleft palates, it seems to me that the cleft palate among profligate women may be due to syphilis, this class being peculiarly liable to it. I will relate the case of a woman in our practice, who came to us with a lesion of the palate, which was distinctly accidental, and we think syphilitic. The insertion of an instrument made it perfectly possible for her to keep her secret. Having known how to speak before, she is enabled to speak perfectly well with the instrument. To the world she is known as "*Miss*," but to us she is known as "*Mrs.* X," for she insists upon posing as a married woman, with the hope of deceiving us. I read Lombroso this summer, and immediately upon my return home there came to me a patient whose model I have here. I may say that this is the only cleft palate that I have had an opportunity to study with these ideas in my mind. I wish to call your attention to the fact that if the cleft of the palate is a sign of degeneracy, she ought to be very degenerate; but that girl is, from every standpoint of mental and moral worth, good enough to be adopted in any family.

*Dr. Fruitnight.*—I was very much interested in Dr. Peterson's paper. It is a very suggestive one. It seems to me the proper application consists in physicians observing these cases in childhood, and I shall certainly observe them more closely hereafter. In this way such degenerates might be put under the proper course as regards mental training, and I think that in this respect the paper is of considerable profit to us. I have no special experience or knowledge on the subject itself.

*Dr. Peck.*—I can make no valuable contribution to the paper, but am glad to speak of its scientific bearing, which commends it to every one of us. To me the subject was comparatively a new one, except in connection with nose and ear practice; but I was very much surprised, on looking up the subject, to find that cleft palate did not exist in idiots, and yet the deformities that were so well explained by Dr. Peterson have been known for many years.

Dr. Down, of the Earlswood Asylum, England, as long ago as 1860, made some valuable contributions in regard to two hundred idiots, and they agree with what has been said to-night. I am sorry Dr. Peterson did not tell us more about the teeth in degenerates, and the narrow width between the teeth and gums in this class of patients. It would have been of great interest to the Society. The average width between the posterior bicuspid teeth of an inch and a half is much less in Down's collection of two hundred idiots, being as low as ten-twelfths of an inch. This was the case whether the patients were young or old, short or tall. A man six feet and one inch high and twenty two years of age presented the narrow space between the posterior bicuspid teeth of one and one-twelfth inches, and between the corresponding gum only five-twelfths of an inch at the widest interval. Age played a significant rôle in Down's table. Of the two hundred idiots under ten years of age there were eight persons; from ten to nineteen years, one hundred and twenty-three persons; from twenty to twenty-nine years, sixty-one persons; from thirty to thirty-nine years, eight persons.

In the quality and quantity of malformation eighty-two showed palates inordinately arched, of whom thirty-four had excessive concavities. There were also varying types of asymmetry in the concavities of the two sides. Seventeen per cent. showed a prominent ridge or keel antero-posteriorly; in seven the palate bones did not meet at all. In none was there a cleft palate; in fact, in six hundred idiots examined by Down, there was no instance of cleft palate.

With such anatomical facts the question arises, Is idiocy a cerebral lesion alone? Is it not due, also, to a want of local nutrition, as shown by the hard palate?

I want to claim the privilege, as a member of the Academy of Medicine, of praising the effort that this Society in particular makes in going outside of its ranks, or to a certain extent outside of its special feature, and bringing out papers of this character. It has been the aim of this Society for a number of years to interlard in its papers special ones which have a bearing on medicine, and it meets with the highest approval of medical men and those who have the interests of the sanitary public at heart. At the time of my membership in the Council of the Academy of Medicine, when I had the honor to represent the Library Committee, dentistry was looked upon as a specialty of the medical art, which is its proper sphere.

I learn that the same opinion now holds. I can only add that scientific work like that of to-night commends itself to every student in medicine.

*Dr. Louise Fiske Bryson.*—I cannot add anything to the discussion, but in examining a large number of children every year, I am struck with the number of dental anomalies. As Dr. Abbott says, the quality of the teeth here exhibited vary materially from the normal. The extremely narrow, highly-pointed palate is one often found associated with deformed teeth and erosions and a form of general malnutrition that points quite markedly to tubercular conditions. That is the only point that I believe has not been discussed.

*Dr. Gouverneur M. Smith.*—I must thank my friend Dr. J. Bond Littig for the invitation to be here this evening. I can warmly corroborate what has just been said by a Fellow of the New York Academy of Medicine in regard to the able manner in which the subject under consideration has been presented and discussed. I know that when the members of the medical profession of this city were building this edifice they wished it to be a grand centre of medical science. Your fraternity is engaged in a collateral calling to our own, and if all the scientific discussions which take place under this roof are conducted in the same careful and painstaking manner which has been shown here this evening, the Academy may well be proud.

Thanking you for calling upon me to take part in the discussion, I would say that I have nothing to offer from my experience which could add interest to what has been already said.

I could not help thinking, however, in connection with the subject of cleft palate, what an important work in one of our great hospitals in New York is now being munificently sustained by reason of an endowment in memory of a most estimable gentleman, a member of one of the old families of this city, who suffered with a congenital cleft palate.[1]

As a boy he was most carefully educated in Europe and America. He graduated at Columbia College, and subsequently at the College of Physicians and Surgeons, later becoming house surgeon in the hospital. His articulation was very materially improved in his eighteenth year by reason of an operation by the late Dr. J. K. Rodgers. Successful as this was, his domestic tastes and studious habits led him to specially cultivate medical science proper, rather than to devote his life to the practice of his calling.

---

[1] Dr. Robert Ray, Jr.

Having become an adept in pathology, shortly after graduating from the hospital he was appointed curator of the hospital cabinet. He assiduously devoted himself to this work, preparing the first printed catalogue of specimens, following the classification of the Guy's Hospital Museum. But the institution was not long to have the benefit of his expert efficiency. In his twenty-eighth year he died from pulmonary tuberculosis.

I knew him in his mature years. He was an earnest student, an accomplished and Christian gentleman. His medical preceptor, the late Dr. John Watson, has appropriately embalmed his estimable character in a biographical sketch.

His devoted father, wishing to perpetuate the memory of his exemplary son, gave to the hospital several lots in this city, with the proviso that the rentals derived from them were to be expended in maintaining the Pathological Museum.

Dr. Thomas M. Markoe, Dr. William T. Bull, Dr. George L. Peabody, and myself constitute the medical committee having supervision of this department. Under the skilful and admirable management of the pathologist, Dr. Frank Ferguson, this large, valuable, and superb museum offers to medical students and medical men a vast field for study. It is an enduring memorial of a young man who died while in his prime, whose life had been tinged with sorrow by reason of an oral defect, and most worthily carries out the views of his honored father.

*Dr. Quinlan.*—My range of observation is very limited in this sphere. I have frequently seen, and know it to be a fact, that many grave diseases, not only of nervous origin, but also of the different organs that are allied to the upper air-tract, have been traced to improper breathing. We know the peculiar facial expression of idiots, the protruding tongue, the hanging of the lower jaw, owing to the depression of the buccinators. Those things have some cause. At birth, and probably in the state before birth, there are physical changes at work that alter certain tissues in the body. One of the most important functions of the body is breathing, and there are numerous follicles that do an immense amount of work. Certain changes take place, and instead of healthy absorption of material, we have a certain growth, and the breathing takes place through the mouth.

This may be a digression from the paper, but notice the effects of this mouth-breathing! I have often made a diagnosis of the location of these parts by inspecting the pharynx. How many children have been relieved! How many members of society have been restored to their families! I remember the case of a little girl with the worst form of chorea I ever saw. She was isolated from her family on account of her feeble health. She was twelve years of age, and the doctor had tried to get her to some institution. I saw the peculiar expression of this class of people, the dry tongue and expression of the eye that characterizes mouth-breathing. I said I thought nothing could be done, and she should be taken to some institution where she could be cared for; but with a view of helping her to breathe better I suggested an operation, which entirely changed the nature of things and restored the child to her family.

A recent instance, mentioned by one of our magazines, is the case of a gentleman who for the past few years had been troubled with melancholia. If he had not had feelings of religion he would have done away with himself. Different operations were suggested by different physicians, and he had undergone a number of operations on almost every part of his body. It was found that one of his nostrils was filled with a polypus, the perpendicular plate of the ethnoid bone was necrosed, and his symptoms were such that the base of the brain was materially affected by the improper circulation of the blood. In a short time he was able to return to society.

Too much attention cannot be paid to this subject. My knowledge of degenerates is narrow, except that in going to some of the institutions for the feeble-minded I notice these peculiar characteristics. Time after time we have seen cases of children who are not able to breathe properly; the nurses will tell you they are irritable and restless, and a mild delirium seizes them. It affects the circulation of the brain, and has this effect on the individual. I thank you for the great privilege and honor of discussing this matter.

*Dr. Peterson.*—I have been very much interested in the discussion, and have read the paper here for the purpose of getting the views of the dentists. In regard to Dr. Abbott's remarks on dental anomalies, I have not paid very much attention to them, except as some of the many stigmata of degeneration. Some of them I am familiar with. As regards microdontism and macrodontism, I have seen a number of cases which were very marked. I know that the neglect of the teeth by most cases of idiocy must lead to a great many abnormalities, but whether any of these are congenital or not I do not know.

Dr. Peirce asked the question as to whether degeneracy was a return to an ancestral type. I do not think so. I think that idea must be abandoned. In the matter of idiocy, Dr. Langdon Down many years ago propounded a classification of idiots according to certain racial types. He spoke of certain cases as being reversions to a lower type of human race. The Mongolian type of idiot resembled the Mongolian race of people, and so on.

In connection with the question of degeneracy, as to whether it is increasing or not, we must remember that besides the fact that has been already mentioned here,—that the degenerates become sterile after a while,—there is also the strong tendency of the hereditary impulse to be normal, that strong impulse of the whole race bearing on each individual to bring him back to a normal type. Those that pass the line and cannot be brought back become sterile in the course of time.

I was much interested in these cases of cleft palate, and I wish the subject could be thoroughly studied from the stand-point of degeneration. I have not been able to find anything in literature in regard to this matter. There are only two or three cases of cleft palate in the collection of four hundred and fifty cases of idiocy and imbecility on Randall's Island. I think it is a question that might be studied with much benefit. Dr. Ottolengui quoted Lombroso a good deal. I look upon him as an extremest in his ideas. He has done a great deal of good in criminal anthropology by calling attention to the degenerative stigmata, and has led others to follow him in investigating this subject; but he himself goes far beyond the facts. I have had the pleasure of meeting him personally and attending some of his criminal clinics, and believe he is too enthusiastic. He is inclined to be a sort of pseudo-anthropologist. His reference to projecting cheek-bones of criminals is rather amusing, because in Italy, where he resides, the projecting cheek-bone is rather rare; but in Scandinavia, where the projecting cheek-bone is common, he would probably change his views.

Dr. Quinlan spoke of mouth-breathing being the cause of a change in the hard palate. In people who are demented I think there is a tendency to mouth-breathing anyhow. In cases of imbecility and even idiocy, where there is not any change in the nasal cavities at all, and no particular change in the palate, you will find that mouth-breathing is a common symptom.

*Dr. Abbott.*—I would move that we pass a vote of thanks to Dr. Peterson for his able paper, and to the other gentlemen who have taken part in this discussion.

Motion carried unanimously.

JOHN I. HART,
*Editor New York Odontological Society.*

